# Evaluation of Vitamin D Metabolism in Patients with Type 1 Diabetes Mellitus in the Setting of Cholecalciferol Treatment

**DOI:** 10.3390/nu12123873

**Published:** 2020-12-18

**Authors:** Alexandra Povaliaeva, Ekaterina Pigarova, Artem Zhukov, Viktor Bogdanov, Larisa Dzeranova, Olga Mel’nikova, Elena Pekareva, Natalya Malysheva, Vitaliy Ioutsi, Larisa Nikankina, Liudmila Rozhinskaya

**Affiliations:** Endocrinology Research Centre, 117036 Moscow, Russia; pigarova.ekaterina@endocrincentr.ru (E.P.); jukov.artem@endocrincentr.ru (A.Z.); bogdanov.viktor@endocrincentr.ru (V.B.); dzeranova.larisa@endocrincentr.ru (L.D.); melnikova.olga@endocrincentr.ru (O.M.); pekareva.elena@endocrincentr.ru (E.P.); malysheva.natalya@endocrincentr.ru (N.M.); ioutsi.vitalij@endocrincentr.ru (V.I.); nikankina.larisa@endocrincentr.ru (L.N.)

**Keywords:** vitamin D, type 1 diabetes mellitus, cholecalciferol, vitamin D-binding protein

## Abstract

In this prospective controlled study, we examined 25 adults with adequately controlled (HbA1c level < 8.0%) type 1 diabetes mellitus (T1DM) and 49 conditionally healthy adults, intending to reveal the diversity of vitamin D metabolism in the setting of cholecalciferol intake at a therapeutic dose. All patients received a single dose (150,000 IU) of cholecalciferol aqueous solution orally. Laboratory assessments including serum vitamin D metabolites (25(OH)D_3_, 25(OH)D_2_, 1,25(OH)_2_D_3_, 3-epi-25(OH)D_3_ and 24,25(OH)_2_D_3_), free 25(OH)D, vitamin D-binding protein (DBP) and parathyroid hormone (PTH) as well as serum and urine biochemical parameters were performed before the intake and on Days 1, 3 and 7 after the administration. The studied groups had no significant differences in baseline parameters except that the patients with diabetes showed higher baseline levels of free 25(OH)D (*p* < 0.05). They also lacked a correlation between the measured and calculated free 25(OH)D in contrast to the patients from the control group (r = 0.41, *p* > 0.05 vs. r = 0.88, *p* < 0.05), possibly due to the glycosylation of binding proteins, which affects the affinity constant for 25(OH)D. The elevation of vitamin D levels after the administration of cholecalciferol was comparable in both groups, with slightly higher 25(OH)D_3_ levels observed in the diabetes group throughout the study since Day 1 (*p* < 0.05). Overall, our data indicate that in patients with adequately controlled T1DM 25(OH)D_3_ levels and the therapeutic response to cholecalciferol is similar to that in healthy individuals.

## 1. Introduction

Sources of vitamin D for humans include exposure to sunlight, food and supplements. Vitamin D_3_ (cholecalciferol) is produced in the skin as a result of exposure to UV radiation. Plant food and some supplements may contain vitamin D in the form of vitamin D_2_ (ergocalciferol). Both of these forms (vitamin D_3_ and D_2_) are conjunctively referred to as vitamin D. In order to gain biological activity, vitamin D further undergoes two stages of hydroxylation: the first one in the C25 position, which takes place in the liver with the formation of 25-hydroxyvitamin D (25(OH)D), and the next in the C1α position. The compound 25(OH)D is universally accepted as the main marker of vitamin D status [1]. The kidneys are the main source of the circulating active form of vitamin D—1,25-dihydroxyvitamin D (1,25(OH)_2_D). Since the discovery of the extrarenal expression of 1α-hydroxylase, the extraskeletal effects of vitamin D due to the local synthesis of the active metabolite have been extensively studied [2,3]. The 24-hydroxylated metabolites of vitamin D are considered as lacking significant physiological roles. The 3-epimer of 25(OH)D is produced by 3-epimerase and is relatively inactive compared with 25(OH)D; however, 3-epi-25(OH)D may be as potent as 1,25(OH)_2_D for parathyroid hormone (PTH) suppression, but there are fewer data on any other biological effects [4].

Vitamin D-binding protein (DBP) is the main transport protein for all vitamin D metabolites: under normal conditions, about 85% of the circulating metabolites are DBP-bound, while albumin binds almost 15% of the remaining metabolites. Less than 1% of the metabolites are found in the bloodstream in the unbound, or free, state [5]. Over the past 10 years, the “free hormone” hypothesis has been actively discussed, which suggests that it is unbound 25(OH)D that exerts many of the nonclassical effects of vitamin D [6].

Data indicating lower 25(OH)D levels in type 1 diabetes mellitus (T1DM) patients comes predominantly from case-control cross-sectional studies [7,8,9] and does not clarify a causal relationship. In this regard, both experimental and clinical prospective studies on the use of vitamin D supplements in T1DM are aimed exclusively at the investigation of the effect of this therapy on the course of the disease [10,11,12,13,14,15,16,17,18,19,20].

The loss of DBP-bound vitamin D in the presence of albuminuria could account for the lower levels of vitamin D in these patients. This is supported by the data of large cross-sectional analyses (NHANES III and NHANES 2001–2006), which showed an association of vitamin D deficiency with an increased rate of albumin excretion and the presence of albuminuria, respectively [21,22]. Urinary DBP levels [23] and vitamin D metabolites (25(OH)D and 1,25(OH)_2_D) [24,25] were higher in individuals with T1DM compared to controls and showed a positive correlation with the duration of diabetes and severity of albuminuria.

The first studies, which were conducted to investigate disorders of vitamin D metabolism in the state of T1DM in the 1980s, proposed that insulin might have a stimulatory effect on enzymes involved in vitamin D metabolism, mostly 25- and 1α-hydroxylase. Hence, insulin deficiency could possibly reduce the activity of these enzymes, leading to decreased levels of some of the vitamin D metabolites [26,27,28]. This hypothesis was supported by more recent studies [29,30]. However, no prospective controlled studies in humans evaluating a broad spectrum of vitamin D metabolites and related biochemical parameters have been performed to date. Revealing the peculiarities of vitamin D metabolism in T1DM is of importance since it could contribute to the more effective compensation of vitamin D deficiency and, possibly, to the control of the underlying disease in these patients, as shown in several intervention studies [31,32,33,34].

The purpose of this study was to reveal distinctive features of cholecalciferol treatment in patients with adequately controlled T1DM compared to conventionally healthy individuals by the dynamic evaluation of serum vitamin D metabolites, free 25(OH)D, transport proteins, and other associated serum and urine biochemical parameters.

## 2. Materials and Methods

### 2.1. Study Population and Design

The study group included 25 adult patients with T1DM, and the control group included 49 conditionally healthy adult individuals. The inclusion criteria were an age from 18 to 60 and HbA1c level < 8.0% for the T1DM group. The exclusion criteria were vitamin D supplementation for 3 months prior to the study; severe obesity (a body mass index >35 kg/m^2^); pregnancy; the presence of granulomatous disease, malabsorption syndrome, or liver failure; a decreased (less than 60 mL/min per 1.73 m^2^); hypercalcemia; allergic reactions to vitamin D medications; and a 25(OH)D level more than 60 ng/mL (determined by immunochemiluminescence analysis). A few patients received therapy that is presumably associated with alterations in vitamin D metabolism within the 3 months preceding the study: antifungal drugs (4 people), oral contraceptives (7 people), anxiolytics (2 people) and antidepressants (3 people). All patients were recruited in the period from July 2019 to September 2020. The study was approved by the Ethics Committee of the Endocrinology Research Centre, Moscow, Russia, on 10 April 2019 (abstract of record No. 6); all patients provided signed informed consent to participate in the study.

All patients received 150,000 IU of an aqueous solution of cholecalciferol (Aquadetrim^®^, Medana Pharma S.A., Sieradz, Poland) orally as a single dose. We obtained blood and urine samples before the intake as well as on Days 1, 3 and 7 after administration. The assessment included serum biochemical parameters (total calcium, albumin, phosphorus, creatinine and magnesium), PTH, DBP, vitamin D metabolites (25(OH)D_3_, 25(OH)D_2_, 1,25(OH)_2_D_3_, 3-epi-25(OH)D_3_ and 24,25(OH)_2_D_3_), free 25(OH)D and urine biochemical parameters (calcium–creatinine and phosphorus–creatinine ratios in spot urine).

### 2.2. Sociodemographic and Anthropometric Data Collection 

At the baseline visit, patients completed a questionnaire aimed at assessing their lifestyles: the presence of unhealthy habits, the physical activity level, a balanced diet (the consumption of dairy products, meat, coffee and soft drinks) and exposure to solar radiation (traveling south and the number of daytime walks in sunny weather in the 3 months preceding study participation). Smoking status was classified as current smoker, former smoker and non-smoker; current and former smokers were collectively referred to as total smokers. A unit of alcohol was defined as a glass of wine, a bottle of beer or a shot of spirits, approximating 10–12 g of ethanol. A serving of dairy products was defined as 100 g of cottage cheese, 200 mL of milk, 125 g of yogurt or 30 g of cheese. Patients’ weights were measured in light indoor clothing with a medical scale to the nearest 100 g, and their heights, with a wall-mounted stadiometer to the nearest centimeter. Body mass index was calculated as weight in kilograms divided by height in meters squared.

### 2.3. Laboratory Measurements

HbA1c levels (reference range, 4.0–6.0%) were measured by high-performance liquid chromatography using an automatic analyzer D10 (BioRad Laboratories, Hercules, CA, USA) and kits from the same manufacturer according to the standard method (certified according to the National Glycohemoglobin Standardization Program).

The total 25(OH)D levels (25(OH)D_2_ +25(OH)D_3_) at the baseline visit were determined by immunochemiluminescence analysis (Liaison, DiaSorin, Saluggia, Italy). PTH levels were evaluated by an electrochemiluminescence immunoassay (ELECSYS, Roche, Basel, Switzerland). Biochemical parameters of the blood serum and urine were assessed using an ARCHITECT c8000 analyzer (Abbott, Chicago, IL, USA) using reagents from the same manufacturer according to standard methods. Serum DBP and free 25(OH)D levels were measured by enzyme-linked immunosorbent assay (ELISA) using commercial kits. The assay used for free 25(OH)D level assessment (DIAsource, ImmunoAssays S.A., Louvain-la-Neuve, Belgium) has <6.2% intra- and inter-assay coefficients of variation (CVs) at levels of 5.8–9.6 pg/mL. The assay used for DBP level assessment (Assaypro, St. Charles, MO, USA) has a 4.0% average intra-assay CV and 9.2% average inter-assay CV.

The levels of vitamin D metabolites (25(OH)D_3_, 25(OH)D_2_, 1,25(OH)2D_3_, 3-epi-25(OH)D_3_ and 24,25(OH)_2_D_3_) in the serum were determined by ultra-high-performance liquid chromatography in combination with tandem mass spectrometry (UPLC-MS/MS) using an in-house-developed method broadly based on the ones published earlier by other research groups [35,36]. A detailed description of the experimental procedure is provided in the Supporting Information (SI). With this technique, the laboratory participates in DEQAS quality assurance program (lab code 2388), and the results fall within the target range for the analysis of 25(OH)D and 1,25(OH)_2_D metabolites in human serum (SI, Figures S1 and S2). All the UPLC-MS/MS measurements were performed after the first successful completion (5/5 samples within the target range) of the DEQAS distributions for both analytes simultaneously. Each batch contained control samples (analytes in blank serum) with both high and low analyte concentrations. The samples were barcoded and randomized prior to the measurements to eliminate analyst-related errors.

The serum samples (3 aliquots) collected at each visit were either transferred directly to the laboratory for biochemical analyses and total 25(OH)D and PTH measurement (1 aliquot) or were stored at − 80 °C, avoiding repeated freeze–thaw cycles, for the measurement of DBP, free 25(OH)D and vitamin D metabolites (2 aliquots).

Baseline free 25(OH)D levels were also calculated using the formula introduced by Bikle et al. [37,38] and repeatedly used in the work of various authors [39,40]. The affinity constant for 25(OH)D and albumin binding (Kalb) used for the calculation was equal to 6 × 10^5^ M^−1^, and the affinity constant for 25(OH)D and DBP binding (KDBP) was equal to 7 × 10^8^ M^−1^.

$$\mathrm{Free}\;25(\mathrm{OH})D\;=\;\frac{\mathrm{total}\;25\left(\mathrm{OH}\right)D}{1+\mathrm{Kalb}*\mathrm{albumin}+\mathrm{KDBP}*\mathrm{DBP}}$$

### 2.4. Statistical Analysis

Statistical analysis was performed using Statistica version 10.0 (StatSoft, Tulsa, OK, USA). All data were analyzed with non-parametric statistics and are expressed as median (interquartile range) unless otherwise specified. The Mann–Whitney U-test and Fisher’s exact test were used for comparisons between two groups. Friedman ANOVA was performed to evaluate changes in indices throughout the study, and pairwise comparisons using the Wilcoxon test with adjustment for multiple comparisons (Bonferroni) were also conducted if the Friedman ANOVA was significant. The Spearman rank correlation method was used to obtain coefficients of correlation among indices. A *p*-value of less than 0.05 was considered statistically significant. 

## 3. Results

The ages, sex ratios, body mass indices and total 25(OH)D levels determined by immunochemiluminescent analysis were equal between the groups (Table 1).

The features of the underlying disease course in the diabetes group are described in Table 2. The median duration of the disease in the diabetes group was 17 years (11; 19). The majority of the patients had intensive glycemic control at the time of participation in the study (median HbA1c level, 7.1% (6.5; 7.6)). All patients received therapy with insulin analogs: about 3/4 of patients received insulin pump therapy, whereas the rest were treated with a basal-bolus insulin regimen. Only two patients (8%) had moderately increased albuminuria, which corresponded to the A2 category; 40% of the patients had nonproliferative diabetic retinopathy, and about 2/3 of the patients had diabetic peripheral neuropathy.

The groups did not differ significantly in the reported levels of physical activity, the amounts of alcohol consumed and dietary habits (Table 3). There was a significantly higher total number of smokers in the control group (39% vs. 12%, *p* = 0.03), while the number of current smokers was comparable between the groups (22% vs. 8%, *p* = 0.2). The number of travelers to the south during the 3 months preceding the study also did not differ significantly (20% in the diabetes group vs. 16% in the control group, *p* = 1.0); however, the individuals in the diabetes group reported more walks in the daytime on a sunny day (9.5 (5; 30) vs. 5 (1; 10), *p* = 0.04).

### 3.1. Baseline Laboratory Evaluation

The detailed results of the laboratory studies are presented in Table 4 and Table 5.

The studied groups had no significant differences in the baseline biochemical parameters of the blood and urine, PTH (Table 4), DBP and vitamin D metabolites (25(OH)D_3_, 25(OH)D_2_, 1,25(OH)_2_D_3_, 3-epi-25(OH)D_3_ and 24,25(OH)_2_D_3_) (Table 5). The median 25(OH)D_3_ levels were similar in both groups (22.3 (18.6; 28.1) ng/mL in the diabetes group vs. 20.5 (14.8; 24.6) ng/mL in the control group, *p* = 0.12) and corresponded to vitamin D insufficiency according to Endocrine Society and Russian Association of Endocrinologists guidelines [1,41]. Only five patients (20%) from the diabetes group and three patients (6%) from the control group had sufficient 25(OH)D_3_ levels (more than 30 ng/mL); the proportions of people with sufficient 25(OH)D_3_ levels did not differ significantly between the groups (*p* = 0.11). Four patients (8%) from the control group were diagnosed with secondary hyperparathyroidism; all participants in the diabetes group had PTH values within the reference interval. 

**Figure 1:**
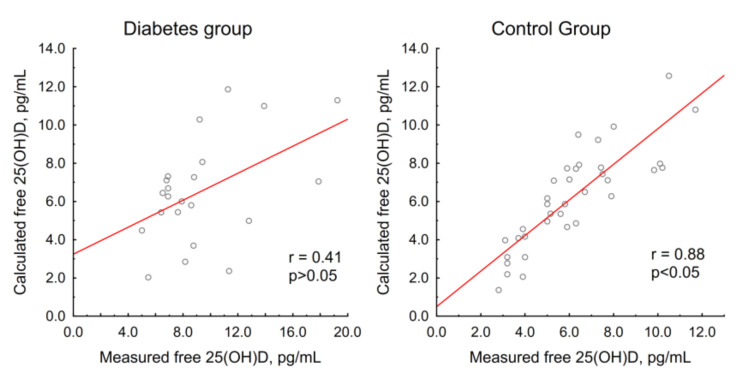
Relationship between measured and calculated free 25(OH)D in groups.

**Figure 2:**
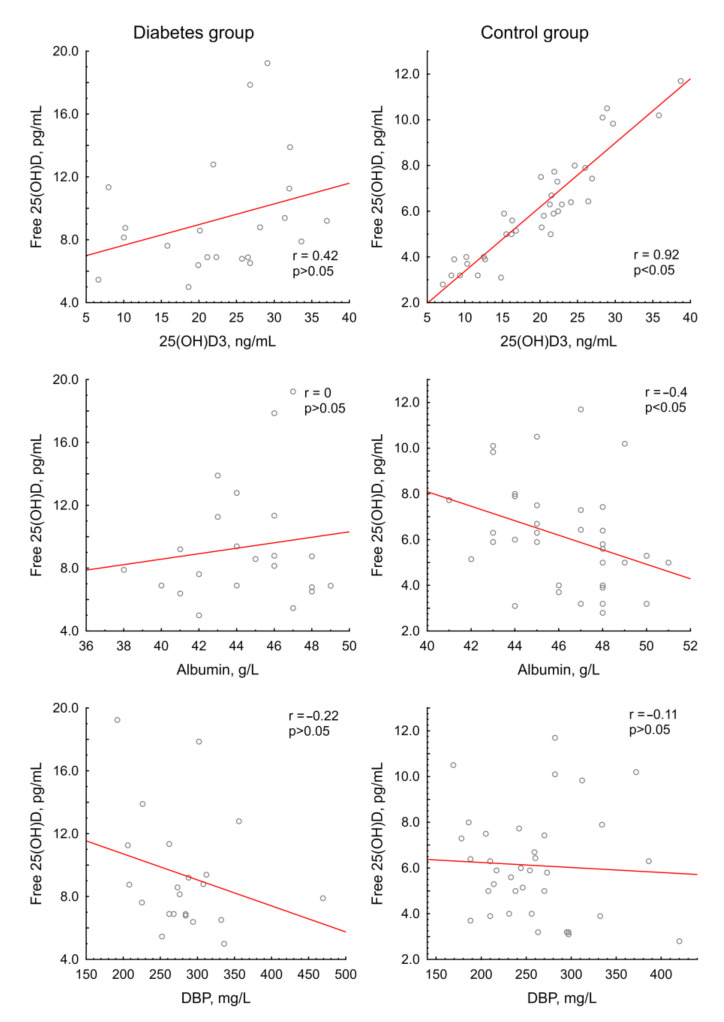
Interrelation of free 25(OH)D and 25(OH)D_3_, transport proteins in groups.

The patients with diabetes showed significantly higher baseline levels of measured free 25(OH)D (8.4 (6.9; 11.3) vs. 5.9 (4.0; 7.5) pg/mL, *p* = 0.0002). The calculated free 25(OH)D levels had a prominent positive correlation with the measured free 25(OH)D levels in the control group (*r* = 0.88, *p* < 0.05), while in the diabetes group, there was only a tendency towards a moderate positive correlation, which did not reach statistical significance (*r* = 0.41, *p* > 0.05) (Figure 1). The measured free 25(OH)D correlated markedly with 25(OH)D_3_ (*r* = 0.92, *p* < 0.05) and serum albumin (*r* = −0.4, *p* < 0.05) in the control group; in the diabetes group, there was no such relationship (*r* = 0.42 and 0, respectively, *p* > 0.05). A correlation between the baseline levels of free 25(OH)D and DBP was not observed in either group (Figure 2).

When analyzing within the diabetes group, no differences were found between the studied parameters depending on the method of therapy received (pump or basal-bolus insulin therapy).

### 3.2. Laboratory Evaluation after Intake of Cholecalciferol

#### 3.2.1. Biochemical Parameters, PTH and DBP

We observed a significant decrease in PTH levels from Day 1 to Day 3, as well as phosphorus levels from Day 3 to Day 7, in the patients with diabetes after they took the drug. A higher phosphorus–creatinine ratio was observed in the diabetes group on Days 3 and 7 compared with the control group.

In the control group, the albumin-corrected calcium and phosphorus levels increased by Day 1, as well as DBP levels, following which DBP decreased to its original values by Day 3. There was also a tendency towards a decrease in PTH by Day 7, of borderline statistical significance (*p* = 0.008). The degree of PTH decrease by Day 7 did not differ between the groups (−4.6 pg/mL in the diabetes group vs. −4.3 pg/mL in the control group, *p* = 0.99).

Only one out of the four patients with secondary hyperparathyroidism had their PTH level normalized by Day 7, which was associated with the normalization of the 25(OH)D_3_ level (39.4 ng/mL on Day 7 vs. 7.1 ng/mL on Day 0). In two patients, an increase in PTH persisted, despite the achieved level of 25(OH)D_3_ being above 30 ng/mL, and in one patient, the received cholecalciferol did not allow reaching the optimal level of vitamin D during the observation period.

One patient from the diabetes group had a transient increase in albumin-corrected calcium to 2.91 mmol/L on Day 1 after taking the drug; in all the other patients, the calcium levels did not exceed the reference range at any point of the study.

#### 3.2.2. Vitamin D Metabolites and Free 25(OH)D

The changes in vitamin D metabolites were generally similar between the groups. The levels of 1,25(OH)_2_D_3_ increased by Day 1, after which they remained stable in both groups throughout the study. As for the other metabolites, we observed an increase in 25(OH)D_3_, 3-epi-25(OH)D_3_ and 24,25(OH)_2_D_3_ levels as well as in the 25(OH)D_3_/24,25(OH)_2_D_3_ ratio by Day 1 in both groups (significantly higher levels of 3-epi-25(OH)D_3_ were noted in the diabetes group on Day 1). By Day 3, the metabolite levels continued to increase, while the 25(OH)D_3_/24,25(OH)_2_D_3_ ratio started decreasing. By Day 7, in both groups, the 24,25(OH)_2_D_3_ levels continued to increase, 3-epi-25(OH)D_3_ levels started decreasing, and the 25(OH)D_3_/24,25(OH)_2_D_3_ ratio decreased further. However, the level of 25(OH)D_3_ remained stable in the patients with diabetes while continuing to increase in the control group. The 25(OH)D_2_ levels did not exceed 0.5 ng/mL in any of the patients throughout the study.

The 25(OH)D_3_ levels were significantly higher on Days 1, 3 and 7 in the diabetes group (*p* < 0.05). Overall, 92% of the patients with diabetes and 84% of the patients from the control group reached 25(OH)D_3_ levels above 30 ng/mL on Day 7 after taking the drug. In both groups, the increase in 25(OH)D_3_ levels negatively correlated with body mass index (*r* = −0.41 in the diabetes group; *r* = −0.46 in the control group; *p* < 0.05).

Free 25(OH)D levels increased significantly by Day 1, continued to increase to Day 3, and decreased by Day 7 in the control group, while in the diabetes group, they remained stable.

## 4. Discussion

The first major finding of the present study consists of the equivalent baseline 25(OH)D_3_ levels between the groups. The patients with diabetes mellitus included in the study had intact kidney function despite a rather long course of the disease (median, 17 years); almost all of them had no albuminuria. Thus, we agree with previous researchers who regarded albuminuria as the principal contributor to the decreased vitamin D levels in patients with diabetes [44,45]. Furthermore, there was little difference in the reported lifestyles between the groups, although the patients with diabetes reported more walking, which may have worked towards a slight improvement in vitamin D status. 

The elevation of vitamin D levels with the dose of cholecalciferol used was comparable in both groups, with slightly higher 25(OH)D_3_ levels observed in the diabetes group throughout the study since Day 1. Nevertheless, a continuous increase in 25(OH)D_3_ throughout the study period was observed in the control group and not in the diabetes group, which may suggest a faster plateau achievement in the diabetes group. The patients with diabetes showed a faster decrease in PTH (as early as Day 3, in contrast to a more gradual decrease throughout the observation period in the control group), which could have been due to higher 3-epi-25(OH)D_3_ levels on Day 1 after taking the drug, as well as higher baseline levels of free 25(OH)D.

The second significant finding was that the patients with diabetes had higher baseline free 25(OH)D levels. As far as the authors are aware, to date, there have been no studies evaluating free 25(OH)D levels in patients with type 1 diabetes mellitus. This effect might be explained by a lower affinity of transport proteins for vitamin D metabolites in diabetes mellitus due to the glycosylation process. Our idea is consistent with the literature data on the altered affinity of glycosylated albumin for some ligands (e.g., several drugs [46,47] and zinc [48]). This hypothesis is also supported by the lack of correlation between the measured and calculated free 25(OH)D in the diabetes group in contrast with the control group. On the other hand, there was no correlation between free 25(OH)D levels and HbA1C or the duration of the disease, but it should be noted that most patients were in a relatively compensated state.

In the healthy individuals, a rapid increase in DBP levels was noted after taking cholecalciferol as early as Day 1, followed by a return to baseline values by Day 3. DBP is considered an acute-phase serum protein and increases during infections or minor injuries [49,50]. Given a high dose of cholecalciferol, a corresponding increase in free 25(OH)D levels is expected, so the observed increase in DBP levels is supposedly aimed at preventing vitamin D toxicity. This is supported by the negative correlation of free 25(OH)D and DBP levels on Days 3 and 7 of the study in the individuals from the control group (r = −0.49 and r = −0.4, *p* < 0.05, respectively). These changes were not observed in the patients with diabetes mellitus: there was no increase in free 25(OH)D or DBP levels, or a correlation between these parameters during the observation period. The foregoing facts could indicate a dysregulation of the mechanism for preventing vitamin D toxicity in patients with diabetes mellitus, and therefore, care should be taken when using high doses of vitamin D in such patients. 

Our study had several limitations: the amount of dietary vitamin D and possible differences in DBP affinity to vitamin D metabolites due to genetic isoforms of DBP [51] were not taken into account. The modest sample size should also be noted. A few patients received therapy that might possibly alter vitamin D metabolism (oral contraceptives, antifungal treatments, antidepressants and anxiolytics).

## 5. Conclusions

Our study indicates that in patients with adequately controlled type 1 diabetes mellitus, in the absence of albuminuria and a corresponding progressive loss of vitamin D metabolites, total 25(OH)D levels and the therapeutic response to cholecalciferol are comparable to those in healthy individuals. The analysis of the vitamin D metabolite levels also suggests that for these patients, insulin deficiency alone does not significantly affect either the regulation of vitamin D metabolism or the activity of the enzymes involved. At the same time, the disturbed balance between the free and protein-bound 25(OH)D fractions observed in the patients with diabetes mellitus suggests that the careful use of high doses of cholecalciferol in the treatment of such patients is necessary. At present, the question remains unresolved regarding the optimal status of vitamin D and the most relevant marker for its assessment, especially under the conditions of various pathological processes. Additional studies are required to determine the constant of the affinity of transport proteins for vitamin D metabolites in diabetes mellitus, as well as the most appropriate marker for assessing the biological functions of vitamin D in this condition.

## Figures and Tables

**Table 1:** General characteristics of the patients at the baseline visit. For a detailed description of the data format, please refer to Section 2.

	Diabetes Group (*n* = 25)	Control Group (*n* = 49)	*p*
Age, years	26.5 (22.7; 36.2)	26.3 (25; 33.5)	0.3
Sex (female, %)	84	80	0.76
Body mass index, kg/m^2^	21.9 (20.7; 25.2)	22.2 (20.1; 26.1)	0.94
25(OH)D total, ng/mL	21.3 (15.9; 29.9)	18.4 (13.3; 25.2)	0.21

**Table 2:** Characteristics of the patients with type 1 diabetes mellitus (T1DM) in terms of the underlying disease.

Parameter	Value
Median duration of T1DM, years	17 (11; 19)
HbA1c, %	7.1 (6.5; 7.6)
Treatment regimen	
Insulin pump (%)	76
Basal-bolus regimen (%)	24
Presence of diabetic complications	
Albuminuria (%)	8
Retinopathy (%)	40
Neuropathy (%)	68

**Table 3:** Questionnaire results.

	Diabetes Group	Control Group	*p*
Current smokers (%)	8	22	0.2
Total smokers (%)	12	39	0.03
Alcohol units, per week	1 (0; 2.5)	1 (0; 2)	0.2
Exercises lasting more than 30 min, per week	3 (2; 5.5)	3 (1.5; 5)	0.44
Dairy product consumption, servings per day	1.5 (1; 2)	1 (0.5; 2)	0.06
Meat dish consumption, portions per week	5 (2; 7)	4.5 (3; 6.5)	0.34
Coffee consumption, cups per week	7 (1; 8)	4.5 (2; 8)	0.61
Soft drinks, mL per week	0 (0; 300)	0 (0; 100)	0.3
Travelers to the south, %	20	16	1.0
Daytime walks in sunny weather, *n*	9.5 (5; 30)	5 (1; 10)	0.04

**Table 4:** Changes in the levels of the biochemical parameters and parathyroid hormone (PTH) during the study.

Laboratory Parameter	Group	Day 0	Day 1	Day 3	Day 7	*p* (Friedman ANOVA)	*p* (Day 0–1)	*p* (Day 1–3)	*p* (Day 3–7)	Reference Range
Total calcium, mmol/L	Diabetes	2.37 (2.33; 2.46)	2.38 (2.33; 2.43)	2.41 (2.36; 2.48)	2.37 (2.31; 2.44)	0.53	-	-	-	2.15–2.55
	Control	2.38 (2.33; 2.45)	2.42 (2.36; 2.45)	2.40 (2.36; 2.46)	2.38 (2.36; 2.45)	0.17	-	-	-	
Albumin-adjusted calcium, mmol/L	Diabetes	2.28 (2.25; 2.32)	2.29 (2.24; 2.32)	2.32 (2.26; 2.36)	2.30 (2.24; 2.35)	0.25	-	-	-	2.15–2.55
	Control	2.26 (2.21; 2.31)	2.29 (2.25; 2.34)	2.30 (2.25; 2.35)	2.28 (2.25; 2.33)	0.007	0.003	0.49	0.17	
Phosphorus, mmol/L	Diabetes	1.19 (1.06; 1.32)	1.23 (1.14; 1.33)	1.27 (1.15; 1.43)	1.17 (1.05; 1.30)	0.001	0.22	0.2	0.001	0.74–1.52
	Control	1.15 (1.06; 1.23)	1.22 (1.10; 1.31)	1.23 (1.18; 1.35)	1.20 (1.12; 1.32)	0.002	0.006	0.21	0.05	
PTH, pg/mL	Diabetes	30.2 (23.5; 37.8)	32.1 (22.2; 38.6)	22.5 (20.9; 26.0) *	25.7 (20.1; 32.4)	0.0003	0.98	0.001	0.49	15–65
	Control	35.9 (28.6; 47.2)	34.3 (23.6; 46.7)	30.5 (21.8; 41.5)	28.6 (22.5; 39.2)	0.002	0.15	0.11	0.55	
Creatinine, μmol/L	Diabetes	71.0 (67.5; 80.2)	71.5 (66.8; 77.9)	73.8 (68.7; 76.6)	74.4 (66.6; 86.1)	0.86	-	-	-	Not applicable
	Control	70.0 (65.9; 74.1)	71.1 (65.7; 74.8)	69.6 (65.7; 73.2)	70.4 (65.5; 75.5)	0.25	-	-	-	
Albumin, g/L	Diabetes	45 (43; 47)	45 (43; 46)	45 (43; 47)	44 (42; 46)	0.65	-	-	-	35–50
	Control	46 (45; 48)	45 (44; 47)	45 (44; 47)	45 (44; 47)	0.66	-	-	-	
Magnesium, mmol/L	Diabetes	0.77 (0.74; 0.81)	0.78 (0.75; 0.83)	0.76 (0.72; 0.80)	0.79 (0.73; 0.82)	0.05	-	-	-	0.7–1.05
	Control	0.80 (0.77; 0.82)	0.79 (0.76; 0.84)	0.79 (0.75; 0.82)	0.79 (0.74; 0.84)	0.23	-	-	-	
Calcium–creatinine ratio, mmol/mmol	Diabetes	0.18 (0.14; 0.28)	0.25 (0.15; 0.31)	0.34 (0.18; 0.46)	0.29 (0.17; 0.46)	0.1	-	-	-	0.1–0.8
	Control	0.28 (0.13; 0.42)	0.31 (0.18; 0.44)	0.31 (0.19; 0.41)	0.30 (0.14; 0.46)	0.79	-	-	-	
Phosphorus–creatinine ratio, mmol/mmol	Diabetes	2.3 (1.8; 3.2)	2.3 (1.7; 3.2)	2.5 (1.9; 3.4) *	2.9 (2.1; 3.4) *	0.19	-	-	-	1.4–3.5
	Control	2.1 (1.3; 2.8)	2.2 (1.0; 2.6)	2.1 (1.4; 2.7)	2.0 (1.2; 2.6)	0.32	-	-	-	

* Significant difference in between-group comparison.

**Table 5:** Changes in the levels of free 25(OH)D, vitamin D-binding protein (DBP) and vitamin D metabolites during the study.

Laboratory Parameter	Group	Day 0	Day 1	Day 3	Day 7	*p* (Friedman ANOVA)	*p* (Day 0–1)	*p* (Day 1–3)	*p* (Day 3–7)	Reference Range
Free 25(OH)D, pg/mL	Diabetes	8.4 (6.9; 11.3) *	10.7 (8.2; 13.8)	14.1 (9.9; 15.9)	13.3 (10.6; 16.3)	0.02	0.09	0.02	0.72	2.4–35 ^1^
	Control	5.9 (4.0; 7.5)	11.2 (8.3; 14.4)	14.0 (10.3; 17)	13.0 (10.0; 15.8)	<0.00001	<0.00001	<0.00001	0.003	
DBP, mg/L	Diabetes	280 (252; 308)	276 (232; 376)	261 (219; 311)	265 (214; 343)	0.03	0.19	0.03	0.65	176–623 ^1^
	Control	255 (212; 289)	300 (246; 338)	259 (218; 287)	275 (240; 313)	0.001	0.001	0.007	0.04	
25(OH)D_3_, ng/mL	Diabetes	22.3 (18.6; 28.1)	35.9 (29; 41.8) *	41.9 (33.4; 46.7) *	41.4 (35.0; 46.3) *	<0.00001	0.00001	0.00004	0.69	>30 ^2^
	Control	20.5 (14.8; 24.6)	31.0 (26.8; 34.4)	34.5 (31.8; 43.1)	36.4 (32.8; 43.1)	<0.00001	<0.000001	<0.000001	0.004	
3-epi-25(OH)D_3_, ng/mL	Diabetes	1.6 (1.3; 2.1)	3.7 (3.0; 4.5) *	4.9 (4.4; 5.8)	4.4 (3.8; 5.1)	<0.00001	0.00001	0.00003	0.00006	Not available
	Control	1.4 (0.9; 1.8)	3.0 (2.5; 3.9)	4.6 (3.9; 5.6)	4.3 (3.4; 5.2)	<0.00001	<0.000001	<0.000001	0.00003	
1,25(OH)_2_D_3_, pg/mL	Diabetes	35 (32; 41)	43 (38; 49)	42 (35; 53)	44 (39; 54)	0.001	0.002	0.33	0.15	25–66 ^3^
	Control	39 (34; 45)	48 (39; 53)	46 (40; 53)	43 (38; 51)	0.001	<0.00001	0.98	0.28	
24,25(OH)_2_D_3_, ng/mL	Diabetes	1.8 (1.4; 3.0)	2.2 (1.6; 3.3)	3.3 (2.2; 4.5)	3.8 (2.6; 4.9)	<0.00001	0.00009	0.00002	0.00005	0.5–5.6 ^3^
	Control	1.7 (0.9; 2.6)	1.9 (1.3; 2.7)	3.0 (2.1; 3.7)	3.5 (2.7; 4.6)	<0.00001	<0.000001	<0.000001	<0.000001	
25(OH)D_3_/24,25(OH)_2_D_3_	Diabetes	12.5 (10.1; 16.0)	16.8 (13.3; 21.9)	11.7 (11.2; 16.0)	10.8 (9.8; 13.8)	<0.00001	0.00001	0.0002	0.00002	7–23 ^3^
	Control	11.9 (9.6; 15.2)	17.0 (12.1; 20.5)	12.3 (10.1; 14.5)	10.4 (8.5; 12.3)	<0.00001	<0.000001	<0.000001	<0.000001	

* Significant difference in between-group comparison. ^1^ Reference ranges are specified according to kit manufacturers’ recommendations. ^2^ Reference range is given for total 25(OH)D according to the clinical guidelines [1,41]; the 25(OH)D_2_ fraction is negligible (<0.5 ng/mL in absolute values) for the purposes of this study. ^3^ Reference ranges are given according to the literature data [42,43].

## Supplementary Materials

The following are available online at https://www.mdpi.com/2072-6643/12/12/3873/s1. Figure S1: Comparison between DEQAS data for 25(OH)D scheme and our lab results; Figure S2: Comparison between DEQAS data for 1,25(OH)_2_D scheme and our lab results; Table S1: MRM transitions used for detection of the vitamin D metabolites.
