# Supplementary material for: Evaluation of Vitamin D Metabolism in Patients with Type 1 Diabetes Mellitus in the Setting of Cholecalciferol Treatment
Source: Nutrients. 2020 Dec 18;12(12):3873. doi: 10.3390/nu12123873 (PMC7767242; doi:10.3390/nu12123873)
Supplement: Supplementary file 1 [file nutrients-12-03873-s001.pdf]

SUPPORTING INFORMATION

Contents

S1. UPLC-MS/MS method for vitamin D_3 metabolites quantification 3

Table S1. MRM transitions used for detection of the vitamin D metabolites 3

S2. Method validation against DEQAS data 4

Figure S1 Comparison between DEQAS data for 25(OH)D scheme and our lab results 4

Figure S2 Comparison between DEQAS data for 1,25$(OH)_2$D scheme and our lab results 4

S1. UPLC-MS/MS method for vitamin D_3 metabolites quantification

50 µl mixture of deuterated internal standards ($25(OH)D_3$-d_6, $1,25(OH)_2D_3$-d_6, 3-epi-$25(OH)D_3$-d_3, $24,25(OH)_2D_3$-d_6) was added to 300 ul of the serum, vortexed and equilibrated for 20 minutes. Then proteins were precipitated by adding 150 µl of 0.05 M ZnSO4 and 500 µl of MeOH, followed by vortexing and centrifugation. Then the resulting liquid was loaded onto Agilent Bond Elut C18 (50 mg, 1ml) cartridges preconditioned with 1ml methanol and 1 ml water. The cartridges were subsequently washed with water followed by a 3:7 methanol/water mixture (1 ml of each), and samples were eluted with 2x300 µl of methanol. The eluate was evaporated to dryness using vacuum centrifuge. Solid extract was derivatized by adding 30 ul of 0.5 mg/ml solution of PTAD (4-phenyl-1,2,4-triazoline-3,5-dione) in acetonitrile. The reaction was quenched after 30 minutes by adding 90 ul of 1:2 methanol/water mixture, transferred to the 384-well plate, and 80 µl of it was injected into the Agilent 1290 Infinity II LC equipped with a 4-channel Flexible pump and Waters Acquity UPLC HSS T3 column (2.1 x 100 mm, particle size 1.8 mm). Gradient elution started with 37% acetonitrile (solvent A), 13% methanol (solvent B) and 50% 0.1% formic acid in water (solvent C),. Then the following program was used: 0-13 min 37% A, 13% B, 50%C; 16 – 18 min 20% A, 20% B, 60% C; 18 – 20 min 100% B; 20 – 23 min 37% A, 13% B, 50%C. Detection was provided by AB Sciex Triple Quad 5500 mass-spectrometer using an ESI source with a capillary voltage of 5500 V and operating in MRM mode (Table S1);

Table S1. *MRM transitions used for detection of the vitamin D metabolites*

Metabolite	Transition type	Q1, Da	Q3, Da	DP, V	CE, V	CXP, V
$24,25(OH)_2D_3$	quantifier	592.3	298.2	145	27	33
	qualifier	592.3	161.2	145	54	16
$24,25(OH)_2D_3$-d_6	IS	598.5	298.2	155	27	31
$1,25(OH)_2D_3$	quantifier	592.3	314.2	130	26	36
	qualifier	574.3	314.2	240	24	39
$1,25(OH)_2D_{3}$-d_6	IS	598.5	314.2	145	25	38
$25(OH)D_3$	quantifier*	558.4	161.1	225	65	18
3-epi-$25(OH)D_3$	quantifier*	558.4	298.3	225	40	36
$25(OH)D_3$-d_6	IS	564.4	298.2	230	30	34
3-epi-$25(OH)D_3$-d_3	IS	561.4	301.1	235	40	35
$25(OH)D_2$	quantifier	605.4	298.2	108	50	32
	qualifier	605.4	161.1	108	42	19

* Quantifiers for $25(OH)D_3$ and 3-epi-$25(OH)D_3$ were used as qualifiers for each other

S2. Method validation against DEQAS

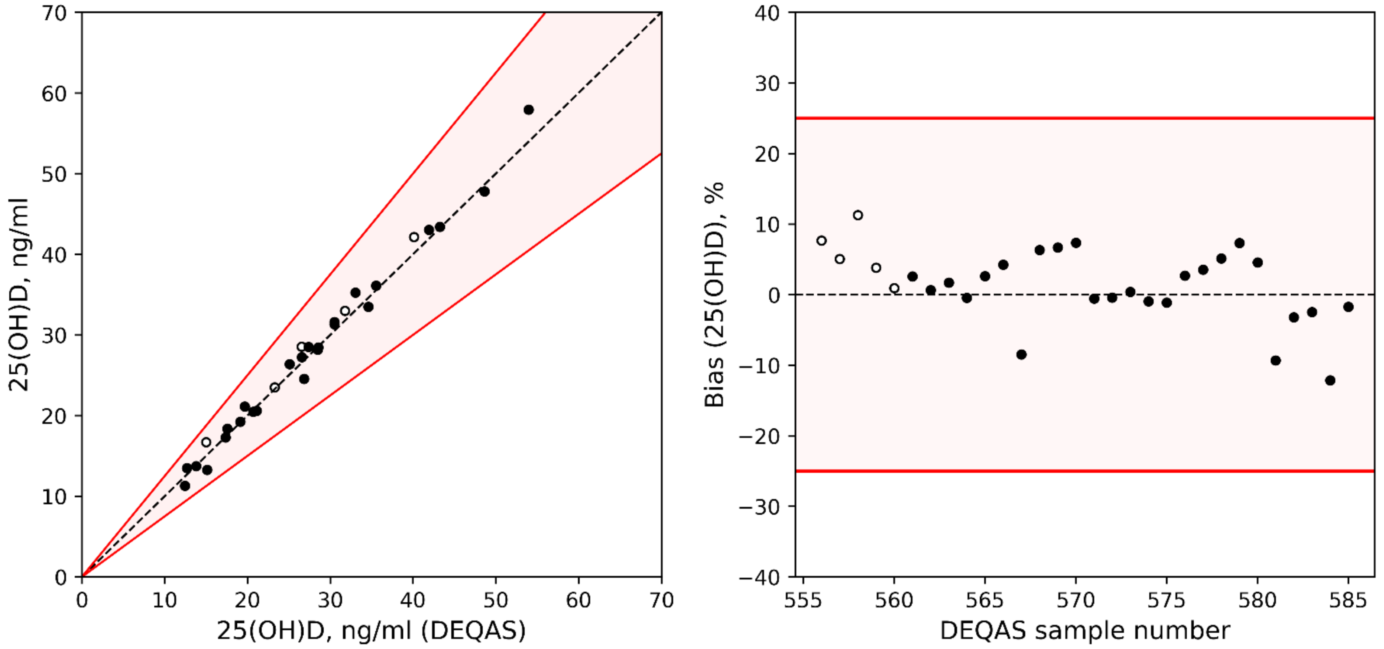

Figure S1 *Comparison between DEQAS data for 25(OH)D scheme and our lab results. Solid dotes denote blind results submitted to DEQAS as lab 2388 prior to publication of the report. Red area indicates DEQAS acceptable range (±25% from the target value)*

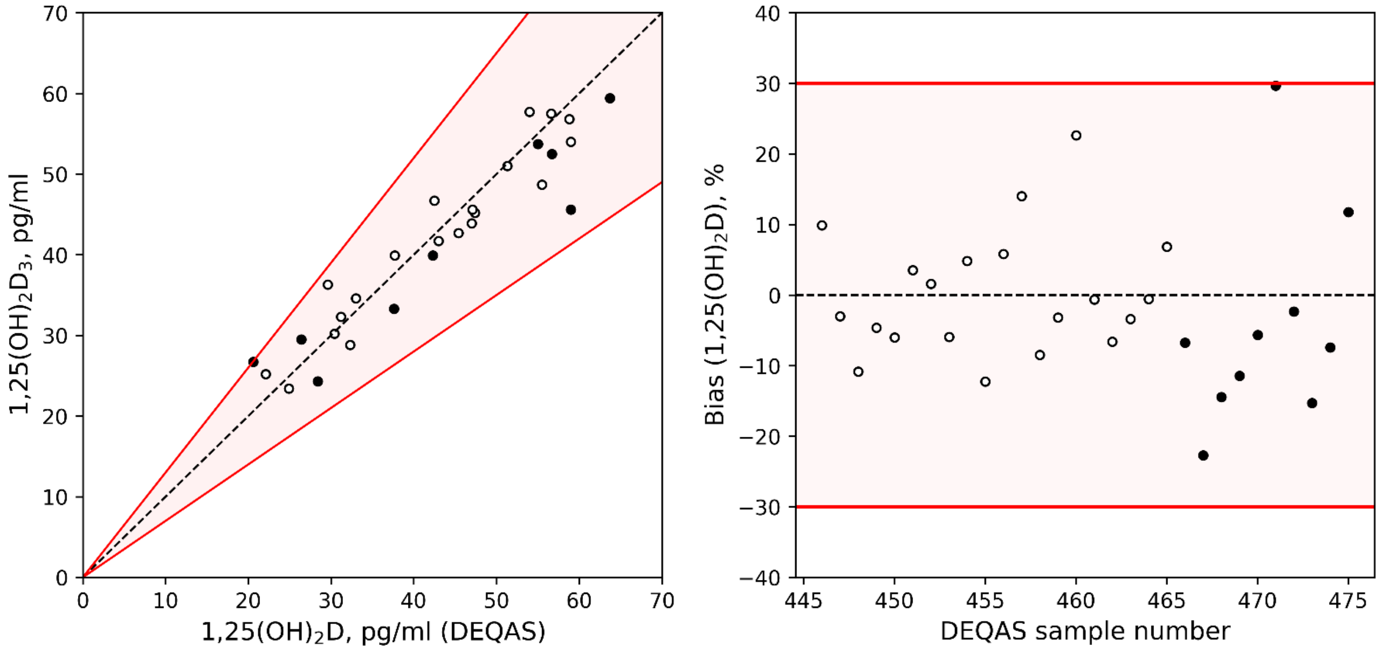

Figure S2 *Comparison between DEQAS data for 1,25(OH)$_2$D scheme and our lab results. Solid dotes denote blind results submitted to DEQAS as lab 2388 prior to publication of the report. Red area indicates DEQAS acceptable range (±30% from the target value)*
